# Summer Dynamics of Microbial Diversity on a Mountain Glacier

**DOI:** 10.1128/msphere.00503-22

**Published:** 2022-11-07

**Authors:** Scott Hotaling, Taylor L. Price, Trinity L. Hamilton

**Affiliations:** a Department of Watershed Sciences, Utah State University, Logan, Utah, USA; b Department of Plant and Microbial Biology and the BioTechnology Institute, University of Minnesota, Saint Paul, Minnesota, USA; National Institute of Advanced Industrial Science and Technology

**Keywords:** alpine glacier, snow algae, glacier biology, cryosphere, biological albedo reduction, alpine

## Abstract

Glaciers are rapidly receding under climate change. A melting cryosphere will dramatically alter global sea levels, carbon cycling, and water resource availability. Glaciers host rich biotic communities that are dominated by microbial diversity, and this biodiversity can impact surface albedo, thereby driving a feedback loop between biodiversity and cryosphere melt. However, the microbial diversity of glacier ecosystems remains largely unknown outside of major ice sheets, particularly from a temporal perspective. Here, we characterized temporal dynamics of bacteria, eukaryotes, and algae on the Paradise Glacier, Mount Rainier, USA, over nine time points spanning the summer melt season. During our study, the glacier surface steadily darkened as seasonal snow melted and darkening agents accumulated until new snow fell in late September. From a community-wide perspective, the bacterial community remained generally constant while eukaryotes and algae exhibited temporal progression and community turnover. Patterns of individual taxonomic groups, however, were highly stochastic. We found little support for our *a priori* prediction that autotroph abundance would peak before heterotrophs. Notably, two different trends in snow algae emerged—an abundant early- and late-season operational taxonomic unit (OTU) with a different midsummer OTU that peaked in August. Overall, our results highlight the need for temporal sampling to clarify microbial diversity on glaciers and that caution should be exercised when interpreting results from single or few time points.

**IMPORTANCE** Microbial diversity on mountain glaciers is an underexplored component of global biodiversity. Microbial presence and activity can also reduce the surface albedo or reflectiveness of glaciers, causing them to absorb more solar radiation and melt faster, which in turn drives more microbial activity. To date, most explorations of microbial diversity in the mountain cryosphere have only included single time points or focused on one microbial community (e.g., bacteria). Here, we performed temporal sampling over a summer melt season for the full microbial community, including bacteria, eukaryotes, and fungi, on the Paradise Glacier, Washington, USA. Over the summer, the bacterial community remained generally constant, whereas eukaryote and algal communities temporally changed through the melt season. Individual taxonomic groups, however, exhibited considerable stochasticity. Overall, our results highlight the need for temporal sampling on glaciers and that caution should be exercised when interpreting results from single or few time points.

## OBSERVATION

Glacier ecosystems are key components of global biodiversity and support diverse, mostly microbial, communities comprising bacteria, photosynthetic algae, and fungi (1 to 3). However, beyond point estimates of biodiversity, seasonal variation of these biota is poorly understood. To date, the majority of biological research on glaciers has focused on establishing baselines of biodiversity (4); understanding the ecophysiology of resident organisms (5), resource availability, and use (6, 7); and clarifying drivers of biological albedo reduction (where pigmented organisms darken the cryosphere and promote melt [2]). Temporal perspectives for all forms of glacier biodiversity remain rare (but see references 8 to 10).

Glacier surfaces are highly dynamic and experience substantial environmental fluxes in space and time. On temperate glaciers, early season “spring” conditions are typically marked by increasing periods of daylight with intense temperature swings and relatively little biological activity. By summer, temperature swings have moderated and biotic activity, including photosynthesis, respiration, and nutrient cycling, nears annual peaks (11). In fall, days shorten, temperatures decrease, and snowfall events limit primary productivity (11).

**FIG 1:**
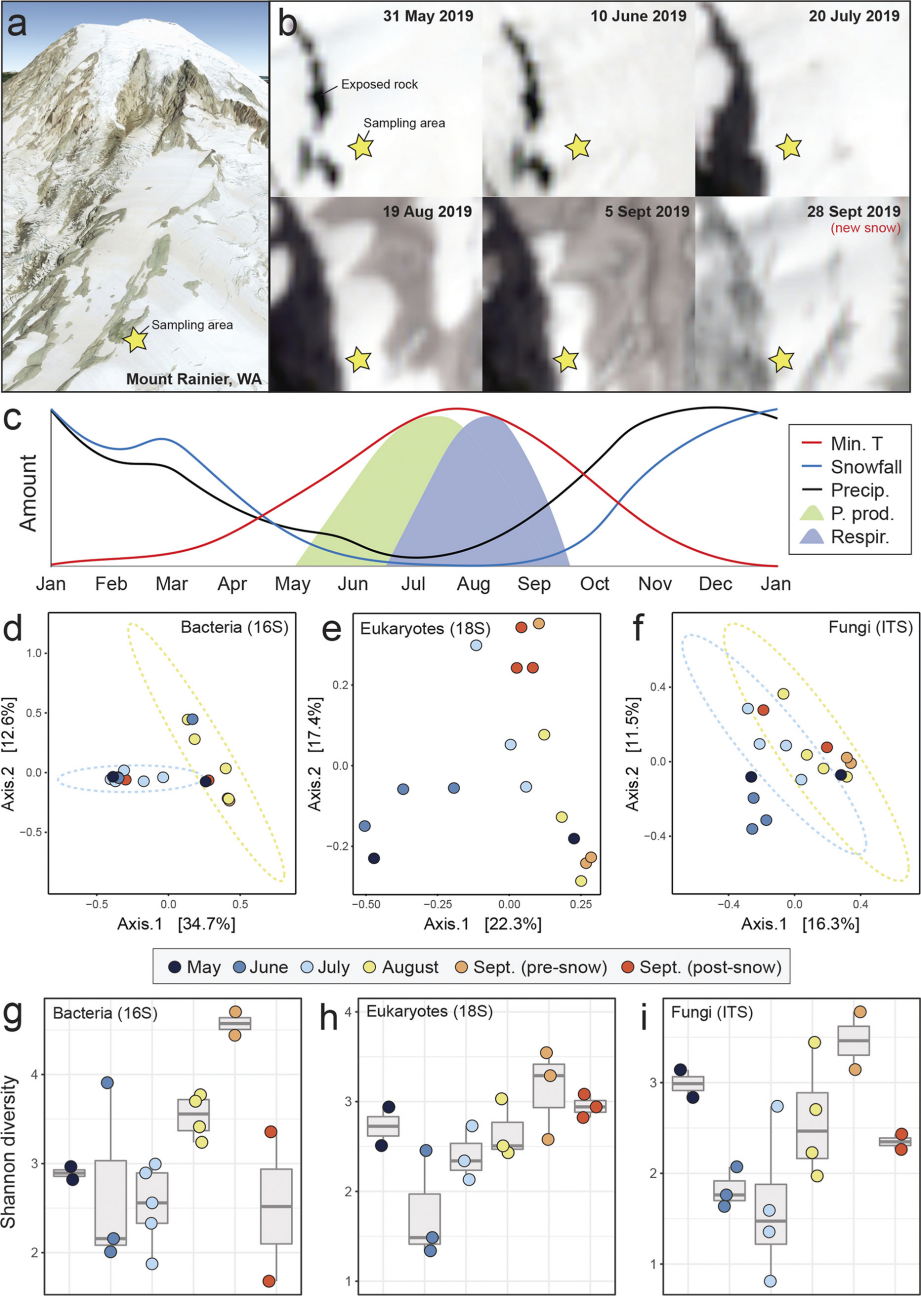
(a) Location of our study site on the Paradise Glacier of Mount Rainier, Washington, USA. Imagery from Google Earth. (b) Sentinel-2 satellite imagery of the study site from late May to September 2019. A fresh snowfall occurred between the final two sampling time points in September. (c) A conceptual image of primary production and heterotroph activity on a temperature mountain glacier over the course of 1 year. Overlaid on this conceptual framework are monthly averages of minimum temperature, average precipitation, and average snowfall for the nearby Paradise Ranger Station (1.655 m) from 1916 to 2016 (data from the Western Regional Climate Center). (d to f) Principal coordinate analysis of community composition based on Bray-Curtis dissimilarity for (d) bacteria, (e) eukaryotes, (f) and fungi (g to i). Shannon diversity through time for the same sampling points and communities: (g) bacteria, (h) eukaryotes, and (i) fungi. Data were binned by sample collection month for visualization (see Table S1 in the supplemental material), and each circle in d to i represents one replicate. After processing, our final sample sizes were the following: 16S rRNA, May: *n* = 2, June: *n* = 3, July: *n* = 5, August: *n* = 4, September: *n* = 2, late September: *n* = 2. For 18S rRNA, May: *n* = 2, June: *n* = 3, July: *n* = 3, August: *n* = 3, September: *n* = 3, late September: *n* = 3. For ITS, May: *n* = 2, June: *n* = 3, July: *n* = 4, August: *n* = 4, September: *n* = 2, late September: *n* = 2.

Here, we present a temporal perspective of microbial community change on the Paradise Glacier, Mount Rainier, Washington, USA (Fig. 1a, b), a temperate alpine glacier that hosts a diverse, representative community of glacier biota. From May to September 2019, we collected triplicate snow samples from ~2,255 m on the eastern margin of the glacier and tracked changes in microbial communities by sequencing 16S and 18S small subunit rRNA and fungal internal transcribed spacer (ITS) amplicons (see Text S1 for extended methods). We expected to uncover a rich biological community on the glacier and evidence of successional dynamics with primary producer abundance peaking early in summer followed by an increase in heterotrophs later in the season.

10.1128/msphere.00503-22.1 TEXT S1 Extended methods. Additional methods used for sample collection, processing, data generation, and statistical analyses are included in a supplemental text file. In addition, a supplemental table (Table S1) is included. Download Text S1, DOCX file, 0.02 MB. Copyright © 2022 Hotaling et al. 2022 Hotaling et al. https://creativecommons.org/licenses/by/4.0/ This content is distributed under the terms of the Creative Commons Attribution 4.0 International license.

10.1128/msphere.00503-22.2 TABLE S1 Date of sample collection and number of reads in each library. Missing libraries or those with a low read count are indicated by grey italic text and were omitted from downstream analyses. Download Table S1, DOCX file, 0.02 MB. Copyright © 2022 Hotaling et al. 2022 Hotaling et al. https://creativecommons.org/licenses/by/4.0/ This content is distributed under the terms of the Creative Commons Attribution 4.0 International license.

During our study, the Paradise Glacier surface darkened as seasonal snow receded, debris accumulated, and biotic processes (e.g., snow algal blooms) transpired until late September, when new snow fell (Fig. 1a and b). In total, we sampled nine time points over 5 months with 21 samples included in downstream analyses (16S: *n* = 18; 18S: *n* = 17; ITS: *n* = 17). Across all samples and time points, we recovered 4,724 bacterial OTUs (16S), 4 archaeal OTUs (16S), 1,246 eukaryotic OTUs (18S), and 3,007 fungal OTUs (ITS). The four archaeal OTUs were rare and thus not considered further. Taxonomic communities were generally not differentiated month to month except for August and July for bacteria and fungi (Fig. 1d, f). Both eukaryotic and fungal communities exhibited seasonal progression (i.e., the amount of time between sampling events appeared to generally scale with community turnover; Fig. 1e, f). In general, alpha diversity increased in all communities from June to September before decreasing following snowfall in late September (Fig. 1g, h, i). Alpha diversity (Shannon’s) was highest for all communities in September (before the snowfall; Fig. 1g, h, i).

**FIG 2:**
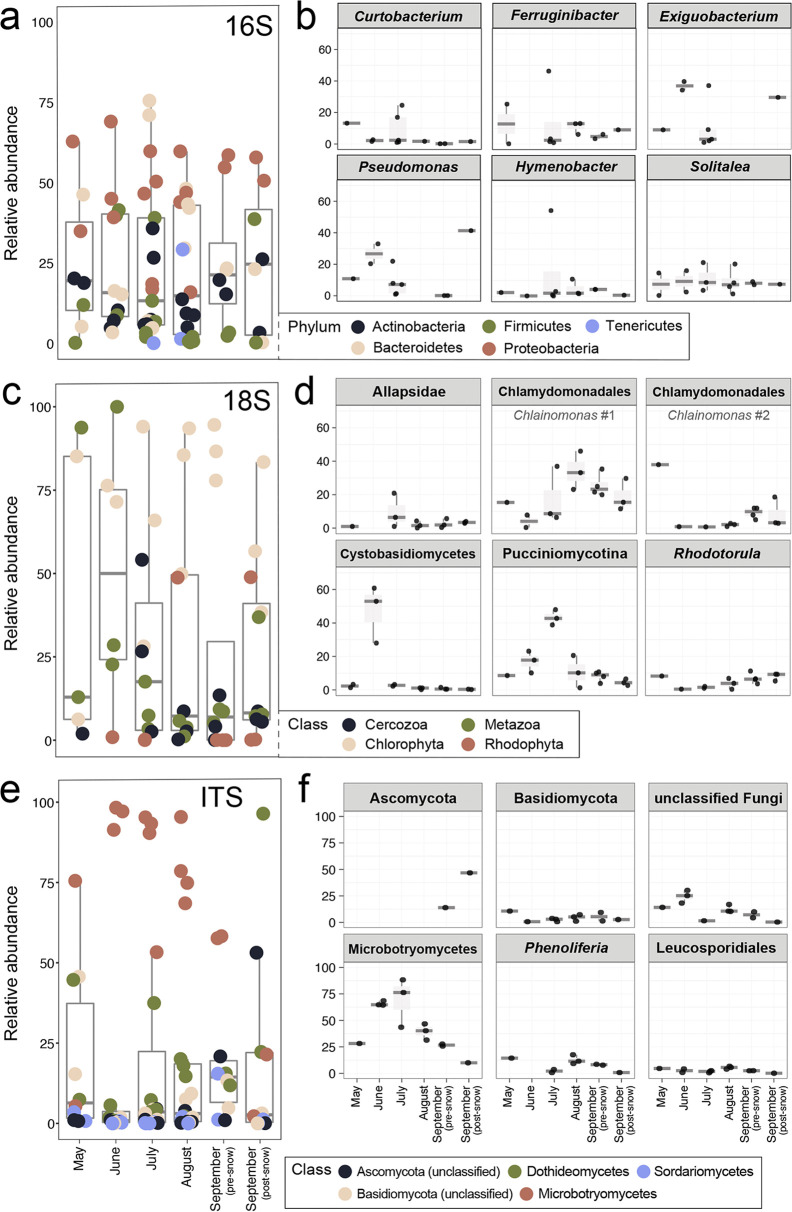
Temporal abundance of common taxonomic groups for each data set overall and broken down for select taxa: (a and b) 16S rRNA, (c and d) 18S rRNA, and (e and f) fungal ITS. Each circle represents one replicate. Taxonomic groups comprising the largest percent relative abundance in each library are shown in a, c, and e. The most abundant operational taxonomic units (OTUs) in each data set are shown in b, d, and f, where taxonomy has been assigned to each OTU at the highest resolution possible (see detailed methods in the supplemental material). Box plots show mean percent relative abundance of the group (a, c, and e) or OTU (b, d, and f). Data sets are binned by month of sample collection except for early and late September, where the first seasonal snow occurred between sampling efforts.

From a taxonomic standpoint, the diversity we recovered aligns with similar studies of snow and ice ecosystems (e.g., reference 10). The most abundant bacterial OTUs we observed were affiliated with Bacteroidetes and Proteobacteria (Fig. 2a and b). Within the Bacteroidetes, OTUs assigned to *Ferruginibacter* and *Solitalea* were most abundant and OTUs assigned to Pseudomonas (Gammaproteobacteria) and *Exiguobacterium* (Bacillota) were also common. For eukaryotes, OTUs assigned to green algae were abundant, including four Chlorophyta OTUs; three were assigned to the snow algae genus *Chlainomonas*, while the fourth belonged to Cyanidiales. Basidiomycota OTUs were prevalent in the fungal data, including 6 of the 10 most abundant OTUs. These 6 OTUs were affiliated with the Microbotryomycetes, including *Phenoliferia* and *Filobasidium* as well as OTUs that could not be classified below the class level.

The abundance of most major bacterial groups fluctuated through time (e.g., Bacteroidetes and Actinobacteria were most abundant in July and less abundant in early September; Fig. 2a; see also Fig. 1d). In contrast, Proteobacteria were abundant in all samples. Algal taxa (phylum Chlorophyta), perhaps the most influential eukaryotes on glaciers (2), were recovered in all samples from all months (Fig. 2c) but were least abundant in June. Algal community composition shifted throughout the summer: abundant *Chlainomonas* OTUs in May and late September were distinct from those recovered in July–September samples (Fig. 2d). For fungi, the relative abundance of sac fungi (Ascomycota) increased in late summer, peaking after the first significant snowfall in September (Fig. 2e). Conversely, the highest abundances of Basidiomycota (the other division that comprises the subkingdom Dikarya alongside Ascomycota) were observed in May, with lower levels from June to September (Fig. 2f).

Broadly, our results support dynamism in both taxonomic composition and abundance of microbial communities on mountain glaciers during the summer melt season. For many groups (e.g., *Ferruginibacter*; Fig. 2b), abundance trends appeared stochastic, or at least not linked to any seasonal dynamics, while others (e.g., Pucciniomycota) exhibited clear directionality across the melt season. Given the resource-poor nature of glacier ecosystems (6), we expected to observe an early-season wave of primary producers followed by an increase in heterotrophs later in the season. Contrary to our expectation, OTUs for snow algal primary producers, particularly *Chlainomonas* within the Chlorophyta, were abundant in all samples except June. Because the same May *Chlainomonas* OTUs increased in abundance in late September, May samples could reflect cells buried from previous years. In contrast, the July–September samples contain *Chlainomonas* OTUs that are distinct from this “resident” community and are perhaps the product of atmospheric input. However, since physical and chemical snow conditions can impact snow algae composition and pigment content (12), which vary seasonally (13), it is also possible that both algal communities are present and local conditions drive the differences we observed. We did observe a decrease in Microbotryomycetes (in the Basidiomycota) and an increase in Ascomycota, fungi which typically favor nutrient-rich niche space (14), in later season samples. Shifts in fungal taxa in response to temperature and nutrients (15) have been linked to resource availability selecting for specific taxa.

With widespread interest in microbial diversity in the cryosphere to better understand carbon cycling, biological albedo reduction, and community ecology of glacier ecosystems (6, 11, 16), it is clear that one or a few estimates of abundance may not reflect broader trends. Thus, our data underscore the need for temporal sampling to ultimately uncover higher-level links between biology and the cryosphere in the mountain cryosphere (2). To realize this potential, such efforts should ideally occur across multiple locations within and among montane regions.
