# Supplementary material for: Summer Dynamics of Microbial Diversity on a Mountain Glacier
Source: mSphere. 2022 Nov 7;7(6):e00503-22. doi: 10.1128/msphere.00503-22 (PMC9769511; doi:10.1128/msphere.00503-22)
Supplement: TEXT S1 [file msphere.00503-22-s0001.docx]

**Supplemental Materials for:**

**Summer dynamics of microbial diversity on a mountain glacier**

Scott Hotaling^1^, Taylor Price^2^, and Trinity L. Hamilton^2^

**Affiliations:**

^1^ Department of Watershed Sciences, Utah State University, Logan, UT, USA

^2^ Department of Plant and Microbial Biology and the BioTechnology Institute, University of Minnesota, Saint Paul, MN, USA

Author 1: Hotaling S ([scott.hotaling1@gmail.com](mailto:scott.hotaling1@gmail.com), ORCID: 0000-0002-5965-0986)

Author 2: Price T ([price793@umn.edu](mailto:price793@umn.edu))

Author 3: Hamilton T ([trinityh@umn.edu](mailto:trinityh@umn.edu), ORCID: 0000-0002-2282-4655)

**Correspondence:**

Scott Hotaling, Department of Watershed Sciences, Utah State University, Logan, UT, USA; Phone: (828) 507-9950; Email: [scott.hotaling1@gmail.com](mailto:scott.hotaling1@gmail.com)

Trinity L. Hamilton, Plant and Microbial Biology, University of Minnesota, St. Paul, USA; Phone: (612) 625-6372; Email: [trinityh@umn.edu](mailto:trinityh@umn.edu)

**Methods:**

**Sample collection and DNA extraction:**

Samples were collected at nine timepoints on the Paradise Glacier from May-September 2019 (Table S1). For each sample, the uppermost ~1 cm of the glacier was scraped with an ethanol-sterilized collection spoon into a 500 mL Whirl-Pak bag or a sterile 15-mL tube until it was filled. Samples were transported on ice and remained frozen until processing. Samples were collected in triplicate. Our intention was to extract and sequence each replicate. Unfortunately, some samples were lost in transit or a low number of reads were recovered for some sequencing libraries (See Table S1, described in more detail below). In the lab, total genomic DNA was extracted using a DNeasy PowerSoil Kit (Qiagen, Carlsbad, CA, USA). The concentration of DNA was determined using a Qubit dsDNA HS Assay kit (Molecular Probes, Eugene, OR, USA) and a Qubit 3.0 Fluorometer (Life Technologies, Carlsbad, CA). All sequenced samples contained more than 1 ng/uL DNA.

**Amplicon sequencing:**

Total DNA for each sample was submitted to the University of Minnesota Genomics Center (UMGC) for amplicon sequencing. Amplicons were sequenced at UMGC using MiSeq Illumina 2 × 300 bp chemistry with the primers 515Ff and 806rB targeting the V4 region of bacterial and archaeal 16S SSU rRNA gene sequences (Caporaso et al., 2012; Apprill et al., 2015), primers E572F and E1009 targeting the V9 region of eukaryotic 18S SSU rRNA gene sequences (Comeau et al., 2011), and primers ITS1F and ITS2R for ITS1 (Tedersoo et al., 2015). UMGC prepared dual indexed Nextera XT DNA libraries following their improved protocol for library preparation which enables detection of taxonomic groups that often go undetected with existing methods (Gohl et al 2016). Each sample was sequenced once.

**Amplicon analysis:**

Post sequence processing was performed using the mothur (ver. 1.45.3) sequence analysis platform (Schloss et al., 2009) following the MiSeq SOP (Kozich et al., 2013) as described previously (Havig and Hamilton, 2019). For 16S and 18S rRNA, read pairs were assembled and resulting contigs with ambiguous bases were removed and trimmed to include only the overlapping regions. For ITS amplicons, we proceeded with only the forward reads by removing ambiguous bases. Chimeras were identified and removed using UCHIME (Edgar et al., 2011) from all the data sets. 16S rRNA amplicons were aligned against the SILVA v138 database and clustered into operational taxonomic units (OTUs) at a sequence similarity of 0.97 with the OptiClust algorithm in mothur. 16S rRNA OTUs classified within mothur using the SILVA database (v138). 18S rRNA amplicons were aligned against the PR^2^ database (ver. 4.12.0) (Guillou et al., 2013).18S rRNA OTUs clustered into OTUs at a sequence similarity of 0.99 using the OptiClust algorithm in mothur. 18S rRNA OTUs and classified within mothur using the PR2 database (v4.12.0). ITS OTUs were clustered into OTUs at a sequence similarity 0.97 using the agc algorithm in mothur. ITS OTUs were classified within Mothur using the UNITE database (v8.2; Abarenkov et al., 2020).

**Statistical analysis:**

All post-mothur processing was carried out in R (ver. 4.0.3; R Core Team, 2018). We anticipated our early season May samples would be low in biomass (based on our observations during sample collection the surface snow was clean/white) and particularly susceptible to contamination from DNA extraction reagents. We also anticipated our samples might contain human 18S rRNA sequences due to the difficulties in maintaining sterile technique during field sample collection. Thus, prior to any analyses, we removed 16S rRNA taxa that are commonly observed in reagent and laboratory contamination (Salter et al., 2014). We also removed 18S rRNA sequences from Mammalia. In our initial analyses of the 18S rRNA amplicon libraries, we also observed large numbers of sequences affiliated with Embryophyceae, particularly fern species. While we can likely attribute these sequences to pollen, we were specifically interested in organisms most likely to be active on snow (or at least have the potential to live the majority of their life on snow) and thus also excluded 18S rRNA sequences affiliated with Embryophyceae. Removal of these contaminants resulted in a low number of remaining reads in some samples (Table S1) and these sample were omitted from downstream analyses. Rarefaction suggested we had sampled the majority of diversity in each library following removal of contaminants.

We calculated diversity indices using vegan (Oksanen et al., 2017) within the Phyloseq package (McMuride and Holnes, 2013) and data were visualized using ggplot2 (Wickham 2016). Data were rarefied to the lowest sequence coverage by random subsampling for richness calculations and transformed using a variance stabilized transformation (Anders and Huber 2010). Diversity was calculated using the Bray-Curtis dissimilarity metric on the transformed data. Because we were interested in community change over time, we binned samples by month of collection for data visualization.

**Data availability:**

All raw sequences are available through the NCBI Sequence Read Archive under BioProject number PRJNA799302.

**References:**

Abarenkov K., Zirk A., Piirmann T., Pöhönen R., Ivanov F., Nilsson, Henrik R.; Kõljalg U. (2020) UNITE mothur release for Fungi. Version 04.02.2020. UNITE Community. https://doi.org/10.15156/BIO/786381

Anders, S., Huber, W. (2010) Differential expression analysis for sequence count data. *Genome Biology*, 11, R106. https://doi.org/10.1186/gb-2010-11-10-r106

Edgar R. C., Haas B. J., Clemente J. C., Quince C., and Knight R. (2011) UCHIME improves sensitivity and speed of chimera detection. *Bioinformatics* 27, 2194–2200. doi: 10.1093/bioinformatics/btr381

Gohl D. M., Vangay P., Garbe J., MacLean A., Hauge A., Becker A., et al. (2016) Systematic improvement of amplicon marker gene methods for increased accuracy in microbiome studies. *Nature* 201:6.

Guillou L., Bachar D., Audic S., Bass D., Berney C., Bittner L., et al. (2013) The Protist Ribosomal Reference database (PR2): A catalog of unicellular eukaryote small sub‐unit rRNA sequences with curated taxonomy. *Nucleic Acids Research*, 41, D597–D604.

Havig, J. R. & Hamilton, T.L. (2019) Cryptic oxygen oases: Hypolithic photosynthesis in hydrothermal areas and implications for Archean surface oxidation. *Front. Earth Sci*. 7:15.

Kozich J. J., Westcott S. L., Baxter N. T., Highlander S. K., Schloss P. D. (2013) Development of a dual-index sequencing strategy and curation pipeline for analyzing amplicon sequence data on the MiSeq Illumina sequencing platform. *Appl. Environ. Microbiol*. 79, 5112–5120. doi: 10.1128/AEM.01043-13

McMurdie P. J. & Holmes S. (2013) phyloseq: an R package for reproducible interactive analysis and graphics of microbiome census data. *PloS One*, 8(4), e61217.

Oksanen J., Blanchet F.G., Friendly M., Kindt R., Legendre P., McGlinn D., et al. (2017) vegan: community ecology package. R package ver- sion 2.4-2. https://cran.r-project.org/web/packages/vegan/index.html.

R Core Team (2018) R: a language and environment for statistical computing. R Foundation for Statistical Computing. <https://www.r-project.org/>

Salter S.J., Cox M.J., Turek E.M., Calus S.T., Cook W.O., Moffatt M.F., Turner P., Parkhill J., Loman N.J., Walker A.W. (2014) Reagent and laboratory contamination can critically impact sequence-based microbiome analyses. *BMC Biol.* 12: 87.

Schloss P.D., Westcott S.L., Ryabin T., Hall J.R., Hartmann M., Hollister E.B., Lesniewski R.A., Oakley B.B., Parks D.H., Robinson C.J. and Sahl J.W. (2009) Introducing mothur: open-source, platform-independent, community-supported software for describing and comparing microbial communities. *Appl. Environ. Microbiol*., 75(23), 7537-7541.

Tedersoo L., Anslan S., Bahram M., Põlme S., Riit T., Liiv I., et al. (2015) Shotgun met- agenomes and multiple primer pair-barcode combinations of amplicons reveal biases in metabarcoding analyses of fungi. *MycoKeys* 10: 1–43.

Wickham H. (2016) ggplot2: Elegant Graphics for Data Analysis. Springer-Verlag New York ISBN 978-3-319-24277-4
