# Supplementary material for: Summer Dynamics of Microbial Diversity on a Mountain Glacier
Source: mSphere. 2022 Nov 7;7(6):e00503-22. doi: 10.1128/msphere.00503-22 (PMC9769511; doi:10.1128/msphere.00503-22)
Supplement: TABLE S1 [file msphere.00503-22-s0002.docx]

**Table S1**. Date of sample collection and number of reads in each library. Missing libraries or those with a low read count are indicated by grey italic text and were omitted from downstream analyses.

|  |  |  | **# of raw reads** | | | **# of read post QC** | | |
| --- | --- | --- | --- | --- | --- | --- | --- | --- |
|  | **Date** | **Rep. #** | **16S** | **18S** | **ITS** | **16S** | **18S** | **ITS** |
| **May** | 11-May-19 | 1 | 50067 | 45050 | 86635 | 27936 | 25546 | 30300 |
|  | 11-May-19 | 2 | 6930 | 40915 | 84480 | 5138 | 5085 | 36531 |
| **June** | 10-Jun-19 | 1 | 8770 | 31106 | 70770 | 7119 | 3107 | 32801 |
|  | 10-Jun-19 | 2 | 13648 | 39675 | 77087 | *360* | 7428 | 37587 |
|  | 17-Jun-19 | 1 | 8632 | 39210 | 71930 | 8127 | 12447 | 27756 |
|  | 17-Jun-19 | 2 | 10217 | 12857 | *793* | 9981 | *530* | *NA* |
| **July** | 8-Jul-19 | 1 | 17025 | 37812 | 8049 | 10754 | *128* | 11172 |
|  | 8-Jul-19 | 2 | 28252 | 42560 | 5645 | 13729 | *62* | *1900* |
|  | 22-Jul-19 | 1 | 12246 | 51575 | 66277 | 10839 | 13600 | 20973 |
|  | 22-Jul-19 | 2 | 17740 | 41942 | 18853 | 11046 | 1469 | 8040 |
|  | 22-Jul-19 | 3 | 19730 | 32398 | 64935 | 9742 | 15685 | 25431 |
| **August** | 4-Aug-19 | 1 | 41651 | 70142 | 62880 | 28701 | 46279 | 23884 |
|  | 4-Aug-19 | 2 | 53136 | 35974 | 76658 | 35152 | 22638 | 28143 |
|  | 23-Aug-19 | 1 | 19663 | 48307 | 34344 | 11836 | *721* | 24652 |
|  | 23-Aug-19 | 2 | 25445 | 53427 | 66476 | 14054 | 14147 | 14345 |
| **September** | 3-Sep-19 | 1 | 58651 | 53776 | 71510 | 27063 | 2861 | 19342 |
| **(pre-snow)** | 3-Sep-19 | 2 | 30093 | 54563 | 79393 | 16566 | 30579 | 22372 |
|  | 3-Sep-19 | 3 | 7584 | 47792 | *682* | *NA* | 39019 | *NA* |
| **Late September** | 23-Sep-19 | 1 | 50525 | 47416 | 103664 | 31527 | 1957 | 52752 |
| **(post-snow)** | 23-Sep-19 | 2 | 37780 | 44179 | 63580 | 21001 | 3038 | 20915 |
|  | 23-Sep-19 | 3 | 6695 | 44652 | *2648* | *362* | 6386 | *NA* |
